# The current status and influencing factors of personal mastery in patients with gout: a cross-sectional study

**DOI:** 10.7717/peerj.21393

**Published:** 2026-06-08

**Authors:** Shuo Cai, Liyun Shang, Yan Lu

**Affiliations:** 1 Department of Hepatobiliary Surgery, The First Affiliated Hospital of Soochow University, Suzhou, China; 2 Department of Oncology, The First Affiliated Hospital of Soochow University, Suzhou, China; 3 School of Nursing, Qingdao University, Qingdao, China

**Keywords:** Gout, Personal mastery, Mindfulness, Pain self-efficacy, Influencing factors

## Abstract

Personal mastery, defined as an individual’s perceived control over life events and environments, plays a significant role in the mental well-being of patients with gout. Our study aimed to assess the level of personal mastery among patients with gout and to analyze its influencing factors. Using convenience sampling, we recruited 234 patients with gout who attended the Shandong Gout Medical Center between October and December 2023. We collected data using the General Information Questionnaire, Personal Mastery Scale, Mindful Attention Awareness Scale, and Pain Self-Efficacy Questionnaire. We used multiple linear regression analysis to examine the factors influencing personal mastery in patients with gout. The total score of personal mastery was (25.3 ± 3.7) in patients with gout. Multiple linear regression analyses showed that education level (*P* < 0.001), monthly income (*P* = 0.019), tophi (*P* = 0.008), number of comorbidities (*P* = 0.014), mindfulness (*P* < 0.001), and pain self-efficacy (*P* < 0.001) were significant predictors of personal mastery in patients with gout. Patients with gout demonstrated moderate levels of personal mastery. Healthcare providers should give particular attention to those with lower education, reduced income, tophi, and multiple comorbidities. Implement targeted interventions to enhance mindfulness and pain self-efficacy, thereby strengthening patients with gout’s personal mastery.

## Introduction

Gout is a form of inflammatory arthritis caused by the deposition of monosodium urate crystals in joints and surrounding tissues (FitzGerald, 2025). It has a global prevalence ranging from <1% to 6.8%, with incidence showing an increasing trend (Dehlin, Jacobsson & Roddy, 2020; Han et al., 2024). As a genetically related chronic disease, gout poses sustained health risks (Asghari et al., 2024; Wang & Jin, 2024). The progressive accumulation of urate crystals can lead to joint structural damage, often resulting in deformity, functional limitation, and recurrent painful flares (Strand et al., 2024). Clinical management follows international guidelines that emphasize controlling acute attacks and maintaining long-term urate-lowering therapy (Khanna et al., 2012; Pascart et al., 2020). However, in practice, patient clinical inertia, related to fears, beliefs, concerns about side effects, low disease knowledge, medication costs, etc., often leads to poor adherence, while physician clinical inertia, related to lack of education focused on treatment targets, etc., often results in inefficient management (Maravic et al., 2018; Phillips et al., 2001). The combined burden of the disease itself and these management challenges significantly impairs mental health, predisposing patients to anxiety, depression, and illness-related uncertainty, which substantially reduces their quality of life (Selvadurai et al., 2024; Zhou et al., 2020).

In light of these self-management challenges and psychological burdens, fostering psychological resources that promote adaptive coping and self-regulation is essential. Personal mastery, defined as an individual’s perceived control over life events and environments, represents a key resource in this regard (Brett & Dubash, 2023). This concept is rooted in the mastery theory proposed by Younger (1991), which posits that individuals confronting illness or adversity are not merely passive but can, through positive processes such as acceptance and change, develop a stronger sense of life mastery and meaning, fostering personal growth (Younger, 1991). As a form of psychological capital, personal mastery not only facilitates stress regulation but is also linked to improved clinical outcomes (Bian et al., 2024; Joshi, Reid & Mindlis, 2025; Neate et al., 2022; Wang et al., 2024). Consistent evidence shows that personal mastery can mitigate anxiety, depression, and psychological distress (Backe et al., 2018; Mereish et al., 2022). Furthermore, personal mastery may empower patients to overcome clinical inertia and engage actively in necessary self-education and disease management (Wang et al., 2024). This is particularly relevant for gout, a condition that demands lifelong self-management strategies, including medication adherence and acute flare control, making personal mastery a crucial asset in its management.

Existing research on personal mastery has predominantly focused on populations such as the elderly, patients with atrial fibrillation, and patients with heart failure (Moreira, Gomes & Pinto, 2022; Wang et al., 2024; Xiang et al., 2024). Within these groups, demographic factors (*e.g.*, income, age, education) and clinical characteristics (*e.g.*, disease duration, comorbidity burden) have been consistently associated with personal mastery (Alharrasi et al., 2024; Drewelies et al., 2017; Kondo et al., 2021; Mitchell et al., 2018; Prasad et al., 2023). However, these relationships remain underexplored in patients with gout. Furthermore, specific psychological mechanisms that may influence personal mastery in patients with gout warrant investigation. Mindfulness, a practice centered on present-moment awareness, acceptance, and non-judgment, closely aligns with the ‘acceptance’ component of mastery theory (Lundgren et al., 2022). By fostering awareness and acceptance of illness-related experiences, mindfulness may enhance adaptive capacity and support personal mastery (Lundgren et al., 2022). Similarly, Pain self-efficacy refers to a patient’s confidence in their ability to manage pain, maintain activities despite pain, and regulate pain-related distress (Ahlstrand et al., 2017). This confidence directly drives proactive coping, aligning with the “change” process in mastery theory and thereby enhancing personal mastery (Ahlstrand et al., 2017). Thus, mindfulness and pain self-efficacy represent theoretically relevant yet understudied psychological correlates in the context of gout. Currently, empirical evidence clarifying their associations with personal mastery in patients with gout is lacking. Investigating these positive psychological constructs could inform the development of novel interventions. Therefore, our study aimed to (1) survey the level of personal mastery in patients with gout, and (2) identify its influencing factors, including demographic, clinical, mindfulness, and pain self-efficacy. The findings are expected to establish a theoretical foundation for designing tailored interventions to improve personal mastery, psychological well-being, and quality of life in patients with gout.

## Materials & Methods

### The subject of the study

This study used a cross-sectional survey design. Using convenience sampling, we recruited patients with gout who attended the Shandong Gout Medical Center as study participants between October 17 and December 30, 2023. This center serves a large regional population across Shandong and neighboring provinces, facilitating recruitment. Eligible participants were required to (1) meet established clinical diagnostic criteria for gout (Neilson et al., 2022) and (2) be aged 18 years or older. Exclusion criteria were: (1) a concurrent life-threatening or end-stage primary organ disease (*e.g.*, advanced metastatic cancer, or severe heart failure (NYHA Class III/IV)); or (2) a severe mental illness or significant cognitive impairment (*e.g.*, major depressive disorder with psychotic features, moderate-to-severe dementia) that would preclude providing reliable informed consent or completing the survey. The sample size was calculated based on the Kendall principle, which recommends 10 to 15 participants per independent variable (Zimmer, Draxler & Debelak, 2023). With 14 independent variables and an anticipated attrition rate of 20%, the target sample size ranged from 175 to 263. The final analysis included 234 patients with gout. The study protocol received approval from the Ethics Committee of Qingdao University Medical School (Approval No. QDU-HEC-2023211), and all procedures were conducted in accordance with the relevant ethical guidelines and regulations.

### Measures

#### General information questionnaire

We used a self-designed questionnaire to collect demographic and clinical characteristic data. The following categorical variables were included: gender, age, education level, monthly income, place of residence, body mass index (BMI), blood uric acid value, duration of illness, number of gouty attacks in the past year, tophi, family history of gout, and number of comorbidities.

#### Personal mastery scale

Personal mastery refers to an individual’s sense of control over their life and environment when facing life events, particularly the challenges of illness. It was assessed using the Personal Mastery Scale (PMS) (Pearlin & Schooler, 1978). The scale is a 7-item, one-dimensional scale. It is rated on a 5-point Likert scale, with scores ranging from 1 to 5, from ‘not at all’ to ‘completely’. Items 1 through 5 are scored negatively, while items 6 and 7 are scored positively. For specific items and scoring details, please refer to Table S1. The scale ranges from a minimum score of 7 to a maximum score of 35, with higher scores indicating greater personal mastery. The Cronbach’s alpha coefficient for the scale is 0.810, and the Cronbach’s alpha coefficient of this scale was 0.849 in our study.

#### Mindful attention awareness scale

Mindfulness was measured using the Mindful Attention Awareness Scale (MAAS) (Brown & Ryan, 2003). The scale is a 15-item, one-dimensional scale. It is rated on a 6-point Likert scale, with scores ranging from 1 to 6, from ‘always’ to ‘never’. The total score ranged from 15 to 90, with higher scores indicating greater mindfulness. The Cronbach’s alpha coefficient for the scale is 0.890, and the Cronbach’s alpha coefficient of this scale in our study was 0.902.

#### Pain self-efficacy questionnaire

Pain self-efficacy was evaluated using the 10-item Pain Self-Efficacy Questionnaire (PSEQ) (Nicholas, 2007). The scale consists of three dimensions, namely, social functioning, pain management, and household/work activities, with 10 items. It is rated on a 7-point Likert scale, with scores ranging from 0 to 6, from ‘not at all confident’ to ‘completely confident’. The total score ranged from 0 to 60, with higher scores indicating greater pain self-efficacy. The Cronbach’s alpha coefficient for the scale is 0.875, and the Cronbach’s alpha coefficient of this scale was 0.909 in our study.

### Data collection

The investigators recruited patients using posters during outpatient visits. They explained the study’s purpose, content, and clinical significance to patients, assuring them that their information would be protected. After obtaining the patient’s written informed consent, the patient began completing the on-site questionnaire. For patients unable to complete the questionnaire independently, the investigators explained any items the patient did not understand and assisted them in answering. Out of 244 questionnaires distributed, 234 were completed and deemed valid for analysis, yielding a response rate of 95.9%.

### Data analysis

All statistical analyses were performed using SPSS software (version 25.0; IBM Corp., Armonk, NY, USA). We used mean and standard deviation to describe the measures that conformed to a normal distribution. Independent-samples *t*-tests and one-way ANOVAs were used to compare levels of personal mastery across demographic and clinical characteristics of patients with gout. Pearson’s correlation analysis was used to analyze mindfulness, pain self-efficacy, and personal mastery. Multiple linear regression was used to analyze multifactorial predictors of personal mastery. Statistical significance is indicated by *P* < 0.05.

**Table 1:** Participants’ demographic and clinical characteristics and differences in personal mastery across characteristics (*n* = 234).

**Variables**	**Category**	**n (%)**	**Personal mastery mean (SD)**	***t/F value***	***P value***
**Gender**	Male	222 (94.9)	25.4 (3.7)	*t*= 2.284	0.023
	Female	12 (5.1)	22.9 (3.0)		
**Age (years)**	18∼44	124 (53.0)	26.4 (3.3)	*F* = 10.847	<0.001
	45∼59	61 (26.1)	24.7 (3.7)		
	60∼74	43 (18.4)	23.3 (3.9)		
	≥75	6 (2.6)	22.7 (4.3)		
**Education level**	Primary school or below	13 (5.6)	21.2 (3.2)	*F* = 25.332	<0.001
	Junior high school	49 (20.9)	22.7 (3.8)		
	High school	38 (16.2)	25.3 (3.5)		
	Junior college or above	134 (57.3)	26.7 (3.0)		
**Monthly income (RMB)**	<2,000	13 (5.6)	21.5 (2.4)	*F* = 18.880	<0.001
	2,000∼3,999	44 (18.8)	22.7 (3.4)		
	4,000∼5,999	63 (26.9)	25.3 (3.4)		
	6,000∼7,999	39 (16.7)	25.5 (3.7)		
	≥8000	75 (32.1)	27.4 (3.0)		
**Place of residence**	Rural	65 (27.8)	23.5 (4.4)	*t* = − 4.656	<0.001
	Urban	169 (72.2)	26.0 (3.2)		
**BMI (kg/m** ^**2**^ **)**	18.50∼23.90 (Normal)	29 (12.4)	24.6 (4.2)	*F* = 2.007	0.137
	24.00∼27.90 (overweight)	121 (51.7)	25.1 (4.0)		
	≥28.00 (obesity)	84 (35.9)	25.9 (3.2)		
**Blood uric acid value (μmol/L)**	≤360.00	65 (27.8)	25.6 (3.8)	*t*= 0.723	0.471
	>360.00	169 (72.2)	25.2 (3.8)		
**Duration of illness (years)**	<1	46 (19.7)	24.9 (3.7)	*F* = 0.818	0.515
	1∼4	69 (29.5)	25.8 (3.4)		
	5∼9	69 (29.5)	25.5 (3.6)		
	10∼14	27 (11.5)	24.7 (4.3)		
	≥15	23 (9.8)	24.7 (4.6)		
**Number of gouty attacks in a year**	0	53 (22.6)	26.9 (3.4)	*F* = 7.714	<0.001
	1∼3	108 (46.2)	25.4 (3.6)		
	4∼6	40 (17.1)	24.9 (3.4)		
	≥7	33 (14.1)	23.1 (4.2)		
**Tophi**	No	125 (53.4)	26.5 (3.3)	*t*= 5.819	<0.001
	Yes	109 (46.6)	23.9 (3.7)		
**Family history of gout**	No	190 (81.2)	25.3 (3.8)	*t* = − 0.037	0.970
	Yes	44 (18.8)	25.3 (3.4)		
**Number of comorbidities**	0	126 (53.8)	26.4 (3.5)	*F* = 12.513	<0.001
	1	49 (20.9)	24.7 (3.8)		
	2	37 (15.8)	24.5 (3.1)		
	≥3	22 (9.4)	21.8 (3.4)		

**Notes.**

SD, standard deviation.

## Results

### Participants’ demographic and clinical characteristics and differences in personal mastery across characteristics

Table 1 shows the characteristics of the participants. A total of 234 patients with gout, aged 18 to 89 (46.2 ± 15.3) years, were included in this study. The majority of participants were male (94.9%, *n* = 222), urban population (72.2%, *n* = 169), and had completed Junior college or higher education (57.3%, *n* = 134). The results showed that gender, age, education level, monthly income, place of residence, tophi, number of gouty attacks in a year, and number of comorbidities were significantly associated with personal mastery (*P* < 0.05). Moreover, BMI, blood uric acid value, duration of illness, and family history of gout were not significantly correlated with personal mastery (*P* > 0.05).

### Correlation of mindfulness, pain self-efficacy, and personal mastery in patients with gout

The results showed that patients with gout had a personal mastery score of 25.3 (3.7). Their mindfulness score was 55.7 (12.5), and their pain self-efficacy score was 35.9 (9.9). The results of Pearson’s correlation analysis showed that mindfulness (*r* = 0.726, *P* < 0.001), pain self-efficacy (*r* = 0.758, *P* < 0.001), and personal mastery were significantly and positively correlated (Table 2).

**Table 2:** Correlation of mindfulness, pain self-efficacy, and personal mastery in patients with gout (*n* = 234).

**Variables**	**Mean (SD)**	**Personal mastery**	**Mindfulness**	**Pain self-efficacy**
**Personal mastery**	25.3 (3.7)	1.000		
**Mindfulness**	55.7 (12.5)	0.726^***^	1.000	
**Pain self-efficacy**	35.9 (9.9)	0.758^***^	0.595^***^	1.000

**Notes.**

SD, standard deviation.

*** *P* < 0.001.

### Multiple linear regression analysis of personal mastery in patients with gout

Variables demonstrating statistical significance in independent-samples *t*-tests, one-way analyses of variance, and correlation analyses were incorporated into regression models. The assignment of the independent variables is shown in Table 3. Regression showed that the variance inflation factors ranged from 1.113 to 1.690, while the tolerance scores ranged from 0.611 to 0.898, indicating no multicollinearity among the variables. The results showed that literacy level (*β* = 0.149, *P* < 0.001), monthly income(*β* = 0.096, *P* = 0.019), tophi (*β* = −0.093, *P* = 0.008), number of comorbidities (*β* = −0.087, *P* = 0.014), mindfulness (*β* = 0.361, *P* < 0.001), and pain self-efficacy (*β* = 0.409, *P* < 0.001) were the factors influencing the personal mastery of patients with gout. These factors collectively explained 75% of the variance. Detailed results are presented in Table 4.

**Table 3:** Independent variable assignment mode.

**Independent variable**	**Assignment**
**Gender**	Male = 1; Female = 2
**Age (years)**	18–44 = 1; 45–59 = 2; 60–74 = 3; ≥75 = 4
**Education level**	Primary school or below = 1; Junior High School = 2; High School = 3; Junior college or above = 4
**Monthly income (RMB)**	<2,000 = 1; 2,000–3,999 = 2; 4,000–5,999 = 3; 6,000–7,999 = 4; ≥8,000 = 5
**Place of residence**	Rural = 1; Urban = 2
**Tophi**	No = 0; Yes = 1
**Number of comorbidities**	0 number = 1; 1 number = 2; 2 number = 3; ≥3 number = 4
**Mindfulness**	Original value
**Pain self-efficacy**	Original value

**Table 4:** Multiple linear regression analysis of personal mastery in patients with gout (*n* = 234).

**Variables**	***B***	***SEB***	*β*	***t value***	***P value***	***95% CI***
**(Constant)**	11.751	0.813	-	14.446	<0.001	10.148, 13.354
**Education level**	0.575	0.156	0.149	3.679	<0.001	0.267, 0.883
**Monthly income (RMB)**	0.284	0.120	0.096	2.366	0.019	0.048, 0.521
**Tophi**	−0.697	0.259	−0.093	−2.691	0.008	−1.207, −0.187
**Number of comorbidities**	−0.320	0.129	−0.087	−2.485	0.014	−0.574, −0.066
**Mindfulness**	0.108	0.013	0.361	8.600	<0.001	0.083, 0.133
**Pain self-efficacy**	0.155	0.016	0.409	9.605	<0.001	0.123, 0.187

**Notes.**

*R*^2^ = 0.756, adjusted *R*^2^ = 0.750, *F* = 117.419, *P* < 0.001.

## Discussion

### The level of personal mastery

Our study found that patients with gout had a mean personal mastery score of 25.3 (SD = 3.7), with scores ranging from 16 to 34. This indicates a moderate level of personal mastery. The score is similar to the average of 25.1 reported in patients with type 2 diabetes (Daniel et al., 2001). Both conditions are chronic and require long-term self-management, which may explain the comparable levels. Several disease-specific challenges may contribute to this moderate level. Gout attacks often occur at night, recur frequently, and are unpredictable. This can lead to a sense of uncertainty and loss of control (Singh, Morlock & Morlock, 2025; Strand et al., 2024). Meanwhile, patients with gout require lifelong disease management in terms of diet and medication. Over the course of long-term self-management, persistent negative psychological states such as maladaptive illness perceptions, depression, stigma, and unhelpful medication beliefs can erode patients’ motivation and engagement in self-management. This disengagement fosters feelings of powerlessness and ultimately weakens their sense of personal mastery (Van der Ven et al., 2025). Moreover, our findings should also be considered within the Chinese cultural context. Traditional beliefs, such as fatalism, and culturally specific fears, such as becoming a burden to one’s family, may shape how patients experience and develop personal mastery. Fatalistic beliefs may hinder acceptance of the illness, while concerns about family responsibility may both motivate and interfere with proactive change. Future cross-cultural research is needed to understand better how personal mastery is formed across different cultural settings.

### Demographic variables and personal mastery

We found that patients with lower levels of education had lower personal mastery, consistent with Kondo’s findings (Kondo et al., 2021). Patients with lower levels of education often lack adequate knowledge of gout (Cai et al., 2024). As a result, they may struggle to recognize and manage their condition effectively, leading to poor disease control and reduced personal mastery (Watson et al., 2024). Therefore, special attention should be given to gout patients with lower levels of education. We recommend implementing structured education programs led by nurses, with collaborative input from pharmacists and physicians (Doherty et al., 2018; Tsiamalou et al., 2023). These programs can use electronic applications, visual aids, simplified language, and the teach-back method to help patients develop practical skills such as medication adherence and dietary adjustment. In this way, patients will gain a clearer understanding of gout, strengthen their self-management abilities, and ultimately reinforce their personal mastery. In addition, our study showed that patients with lower monthly incomes also had lower levels of personal mastery, which aligns with Koltai’s findings (Koltai, Bierman & Schieman, 2018). Patients with lower incomes often face a heavier financial burden and may lose hope in life, both of which contribute to diminished personal mastery (Xiang et al., 2024). To address this, we suggest that health authorities continue to improve the insurance system by including commonly used gout medications such as allopurinol and febuxostat in outpatient chronic disease coverage. Pilot programs such as out-of-pocket expense caps or medication subsidies should also be explored for low-income patient groups. These measures will help alleviate financial burden and strengthen patients’ sense of personal mastery.

### Clinical characteristics and personal mastery

We found that patients with tophi had lower personal mastery than those without. Tophi can cause joint deformities, leading to progressive loss of joint function and reduced mobility (Jiang et al., 2024). This loss of mobility disrupts daily life and diminishes patients’ sense of control over themselves and their surroundings (Coulshed et al., 2020). We also found that patients with more comorbidities reported lower personal mastery, consistent with Alharrasi’s findings (Alharrasi et al., 2024). Gout is closely associated with conditions such as hypertension and coronary heart disease (Dumusc & So, 2024). Comorbidities complicate treatment and require patients to manage multiple conditions simultaneously, often accompanied by a higher burden of medications, increased risk of side effects, and more frequent follow-up visits. This can divert attention away from gout self-management and reduce personal mastery (Walsh et al., 2016). Furthermore, the relationship between tophi, comorbidities, and personal mastery may be bidirectional. Patients with lower personal mastery often struggle with self-management, which may in turn contribute to the development of tophi and other complications, creating a vicious cycle. Therefore, in clinical practice, healthcare providers should pay particular attention to gout patients with tophi or multiple comorbidities. We recommend implementing comprehensive support measures, including standardized urate-lowering therapy, comorbidity management, and patient education, to help patients manage their overall health more effectively and break the vicious cycle between disease progression and diminished sense of personal mastery.

### Mindfulness and personal mastery

We found that mindfulness was positively associated with personal mastery in patients with gout. This finding is consistent with the theory of mastery, which identifies “acceptance” as a key psychological process in achieving personal mastery (Younger, 1991). Mindfulness, a practice centered on acceptance and non-judgment, helps patients come to terms with the reality of their illness (Lundgren et al., 2022). Patients with higher levels of mindfulness are better able to focus on the present moment. They observe and accept the physical and emotional experiences associated with their disease with a calm and open attitude, rather than avoiding or resisting them (Pang et al., 2024). This acceptance-oriented mindset allows patients to gain clearer insight into their condition, regulate their emotions more effectively, and engage in disease management. In turn, these capacities lay the groundwork for stronger personal mastery (Pang et al., 2024; Zeng et al., 2025). In contrast, patients with low mindfulness often struggle to achieve acceptance. They may avoid or exaggerate pain, in part because they have difficulty accurately perceiving their internal state (Lam et al., 2024). This failure to accept their illness directly impedes the development of personal mastery (Kim et al., 2025; Uritani et al., 2020). Previous studies have shown that mindfulness training can enhance personal mastery by strengthening patients’ capacity for acceptance (Li et al., 2018; Lundgren et al., 2022). Therefore, we recommend that healthcare providers incorporate mindfulness training into routine health management plans as a key psychological intervention to support acceptance and improve their sense of personal mastery.

### Pain self-efficacy and personal mastery

We found that pain self-efficacy was positively associated with personal mastery in patients with gout. This relationship can be understood within the framework of mastery theory, in which pain self-efficacy reflects the “change” process described by Younger (1991). Patients with higher pain self-efficacy believe in their ability to cope with pain and its distress (Ferrari et al., 2019). This confidence motivates them to manage their pain and seek effective strategies actively (Du et al., 2018; Schumann et al., 2022). This proactive coping represents the behavioral manifestation of the “change” process. When patients observe improvements in their pain status through their own efforts, their personal mastery consequently strengthens (Englid, Jirwe & Conte, 2023). Conversely, patients with low pain self-efficacy exhibit insufficient motivation and capacity for change, often adopting passive avoidance strategies toward pain (Li et al., 2022). They feel powerless in managing their pain, and this sense of helplessness directly impedes the development of personal mastery (Wang et al., 2025). Therefore, healthcare providers should focus on enhancing pain self-efficacy in patients with gout. By sharing success stories, offering encouragement, and using other supportive strategies, they can help patients build confidence in their ability to manage pain, thereby strengthening their sense of personal mastery.

### Study strengths and limitations

This is the first study on the current situation and factors influencing personal mastery in patients with gout. The study’s results provide a scientific foundation for developing and implementing individualized intervention programs to improve patients’ personal mastery. It is of positive significance for improving the theoretical framework of chronic disease self-management. In addition, the measurement tools used in the study, including the Personal Mastery Scale, Mindful Attention Awareness Scale, and Pain Self-Efficacy Questionnaire, have been widely used in China to ensure their reliability and validity. However, several limitations should be acknowledged. First, the cross-sectional design precludes causal inference about the relationship between personal mastery and its associated factors. Longitudinal studies are needed to establish temporal relationships. Second, the study was conducted in Qingdao, a relatively developed coastal city in China. The sample was predominantly urban (72.2%) and well educated (57.3%), which may limit the generalizability of our findings to regions with different sociodemographic profiles, such as rural or less developed areas. In addition, although the high proportion of male participants (94.9%) reflects the epidemiology of gout, caution is warranted when extrapolating these results to female patients. Third, while we collected key clinical indicators, some factors were not systematically assessed, including detailed alcohol consumption history and specific comorbid conditions (*e.g.*, coronary artery disease, chronic heart failure, chronic kidney disease, and type 2 diabetes with micro or macro vascular complications). Future studies should adopt a multicenter design and intentionally recruit participants from diverse geographic, socioeconomic, and educational backgrounds, including a larger proportion of female patients. Incorporating standardized assessments of key lifestyle behaviors and conducting cross-cultural comparisons would further enhance the generalizability and comprehensiveness of the findings.

## Conclusions

Patients with gout in our study demonstrated moderate personal mastery, suggesting room for further improvement. Education level, monthly income, tophi, number of comorbidities, mindfulness, and pain self-efficacy were the core factors influencing personal mastery in patients with gout. Healthcare providers should deliver targeted health education to enhance patients’ health literacy and strengthen their ability to manage tophus and associated complications. Additionally, interventions that reinforce mindfulness and pain self-efficacy should be sustained to further improve the sense of personal mastery in patients with gout.

## Supplemental Information

10.7717/peerj.21393/supp-1 Supplemental Information 1 Personal Mastery Scale (PMS).

10.7717/peerj.21393/supp-2 Supplemental Information 2 Supplemental Data.

10.7717/peerj.21393/supp-3 Supplemental Information 3 Codebook.

10.7717/peerj.21393/supp-4 Supplemental Information 4 STROBE checklist.

## Additional Information and Declarations

### Data Availability

The following information was supplied regarding data availability: The data is available in the - Supplementary Files. The data is available at FigShare: - Cai, Shuo (2026). S1 dataset. figshare. Dataset. https://doi.org/10.6084/m9.figshare.28731173.v1

## References

[ref-1] Ahlstrand I, Vaz S, Falkmer T, Thyberg I, Björk M (2017). Self-efficacy and pain acceptance as mediators of the relationship between pain and performance of valued life activities in women and men with rheumatoid arthritis. Clinical Rehabilitation.

[ref-2] Alharrasi M, Al-Noumani H, Al-Ghassani A, Al-Jadidi S, Al-Maskari M, Al-Zakwani I (2024). Perceived control attitude among heart failure patients in Oman: a multicenter study. Scientific Reports.

[ref-3] Asghari KM, Zahmatyar M, Seyedi F, Motamedi A, Zolfi M, Alamdary SJ, Fazlollahi A, Shamekh A, Mousavi SE, Nejadghaderi SA, Mohammadinasab R, Ghazi-Sha’rbaf J, Karamzad N, Sullman MJM, Kolahi AA, Safiri S (2024). Gout: global epidemiology, risk factors, comorbidities and complications: a narrative review. BMC Musculoskeletal Disorders.

[ref-4] Backe IF, Patil GG, Nes RB, Clench-Aas J (2018). The relationship between physical functional limitations, and psychological distress: considering a possible mediating role of pain, social support and sense of mastery. SSM—Population Health.

[ref-5] Bian Z, Xu R, Shang B, Lv F, Sun W, Li Q, Gong Y, Luo C (2024). Associations between anxiety, depression, and personal mastery in community-dwelling older adults: a network-based analysis. BMC Psychiatry.

[ref-6] Brett G, Dubash S (2023). The sociocognitive origins of personal mastery. Journal of Health and Social Behavior.

[ref-7] Brown KW, Ryan RM (2003). The benefits of being present: mindfulness and its role in psychological well-being. Journal of Personality and Social Psychology.

[ref-8] Cai S, Hu D, Wang D, Zhao J, Du H, Wang A, Song Y (2024). Health literacy in patients with gout: a latent profile analysis. PLOS ONE.

[ref-9] Coulshed A, Nguyen AD, Stocker SL, Day RO (2020). Australian patient perspectives on the impact of gout. International Journal of Rheumatic Diseases.

[ref-10] Daniel M, Rowley KG, Herbert CP, O’Dea K, Green LW (2001). Lipids and psychosocial status in aboriginal persons with and at risk for type 2 diabetes: implications for tertiary prevention. Patient Education and Counseling.

[ref-11] Dehlin M, Jacobsson L, Roddy E (2020). Global epidemiology of gout: prevalence, incidence, treatment patterns and risk factors. Nature Reviews Rheumatology.

[ref-12] Doherty M, Jenkins W, Richardson H, Sarmanova A, Abhishek A, Ashton D, Barclay C, Doherty S, Duley L, Hatton R, Rees F, Stevenson M, Zhang W (2018). Efficacy and cost-effectiveness of nurse-led care involving education and engagement of patients and a treat-to-target urate-lowering strategy *versus* usual care for gout: a randomised controlled trial. Lancet.

[ref-13] Drewelies J, Wagner J, Tesch-Römer C, Heckhausen J, Gerstorf D (2017). Perceived control across the second half of life: the role of physical health and social integration. Psychology and Aging.

[ref-14] Du S, Hu L, Bai Y, Dong J, Jin S, Zhang H, Zhu Y (2018). The influence of self-efficacy, fear-avoidance belief, and coping styles on quality of life for chinese patients with chronic nonspecific low back pain: a multisite cross-sectional study. Pain Practice.

[ref-15] Dumusc A, So A (2024). Advances in treatment of hyperuricemia and gout. Therapeutische Umschau.

[ref-16] Englid MB, Jirwe M, Conte H (2023). Perioperative comfort and discomfort: transitioning from epidural to oral pain treatment after pancreas surgery: a qualitative study. Journal of PeriAnesthesia Nursing.

[ref-17] Ferrari S, Vanti C, Pellizzer M, Dozza L, Monticone M, Pillastrini P (2019). Is there a relationship between self-efficacy, disability, pain and sociodemographic characteristics in chronic low back pain? A multicenter retrospective analysis. Archives of Physiotherapy.

[ref-18] FitzGerald JD (2025). Gout. Annals of Internal Medicine.

[ref-19] Han T, Chen W, Qiu X, Wang W (2024). Epidemiology of gout—global burden of disease research from 1990 to 2019 and future trend predictions. Therapeutic Advances in Endocrinology and Metabolism.

[ref-20] Jiang X, Li A, Hao W, Yang C, Wang H, Deng W (2024). Limb salvage and systemic management of gouty tophi: Case series. Medicine.

[ref-21] Joshi S, Reid MC, Mindlis I (2025). Illness intrusiveness, perceived control, and quality of life in older adults with arthritis and multimorbidity. Clinical Gerontologist.

[ref-22] Khanna D, Fitzgerald JD, Khanna PP, Bae S, Singh MK, Neogi T, Pillinger MH, Merill J, Lee S, Prakash S, Kaldas M, Gogia M, Perez-Ruiz F, Taylor W, Lioté F, Choi H, Singh JA, Dalbeth N, Kaplan S, Niyyar V, Jones D, Yarows SA, Roessler B, Kerr G, King C, Levy G, Furst DE, Edwards NL, Mandell B, Schumacher HR, Robbins M, Wenger N, Terkeltaub R (2012). 2012 American College of Rheumatology guidelines for management of gout. Part 1: systematic nonpharmacologic and pharmacologic therapeutic approaches to hyperuricemia. Arthritis Care and Research.

[ref-23] Kim ES, Chen Y, Hong JH, Lachman ME, VanderWeele TJ (2025). Mastering the canvas of life: identifying the antecedents of sense of control using a lagged exposure-wide approach. Applied Psychology: Health and Well-Being.

[ref-24] Koltai J, Bierman A, Schieman S (2018). Financial circumstances, mastery, and mental health: taking unobserved time-stable influences into account. Social Science and Medicine.

[ref-25] Kondo A, Oki T, Otaki A, Abuliezi R, Eckhardt AL (2021). Factors related to perceived control and health-related quality of life of patients after acute coronary syndrome during admission and after discharge. Japan Journal of Nursing Science.

[ref-26] Lam CN, Larach DB, Chou CP, Black DS (2024). Mindful attention is inversely associated with pain *via* mediation by psychological distress in orthopedic patients. Pain Medicine.

[ref-27] Li Q, Chen Y, Välimäki M, Long Q, Yang J, Guo J (2022). The association between general self-efficacy and depressive symptoms in people with type 2 diabetes mellitus: the mediating role of coping styles preference. Psychology Research and Behavior Management.

[ref-28] Li Y, Liu F, Zhang Q, Liu X, Wei P (2018). The effect of mindfulness training on proactive and reactive cognitive control. Frontiers in Psychology.

[ref-29] Lundgren O, Garvin P, Nilsson L, Tornerefelt V, Andersson G, Kristenson M, Jonasson L (2022). Mindfulness-based stress reduction for coronary artery disease patients: potential improvements in mastery and depressive symptoms. Journal of Clinical Psychology in Medical Settings.

[ref-30] Maravic M, Hincapie N, Pilet S, Flipo RM, Lioté F (2018). Persistent clinical inertia in gout in 2014: an observational French longitudinal patient database study. Joint Bone Spine.

[ref-31] Mereish EH, Parra LA, Watson RJ, Fish JN (2022). Subtle and intersectional minority stress and depressive symptoms among sexual and gender minority adolescents of color: mediating role of self-esteem and sense of mastery. Prevention Science.

[ref-32] Mitchell UA, Ailshire JA, Brown LL, Levine ME, Crimmins EM (2018). Education and psychosocial functioning among older adults: 4-year change in sense of control and hopelessness. The Journals of Gerontology. Series B, Psychological Sciences and Social Sciences.

[ref-33] Moreira MTC, Gomes CS, Pinto JM (2022). Influence of personal mastery on mobility disability among older adults: a systematic review. Archives of Gerontology and Geriatrics.

[ref-34] Neate S, Humam A, Nag N, Jelinek GA, Simpson-Yap S (2022). Greater mastery is associated with lower depression risk in a large international cohort of people with multiple sclerosis over 2.5 years. Quality of Life Research.

[ref-35] Neilson J, Bonnon A, Dickson A, Roddy E (2022). Gout: diagnosis and management-summary of NICE guidance. Bmj.

[ref-36] Nicholas MK (2007). The pain self-efficacy questionnaire: taking pain into account. European Journal of Pain.

[ref-37] Pang Y, Tse B, Liu W, Yang Q (2024). The relationship between mindfulness and cognitive reappraisal: the mediating role of emotional and interoceptive awareness. Cognitive Processing.

[ref-38] Pascart T, Latourte A, Flipo RM, Chalès G, Coblentz-Baumann L, Cohen-Solal A, Ea HK, Grichy J, Letavernier E, Lioté F, Ottaviani S, Sigwalt P, Vandecandelaere G, Richette P, Bardin T (2020). 2020 recommendations from the French Society of Rheumatology for the management of gout: urate-lowering therapy. Joint Bone Spine.

[ref-39] Pearlin LI, Schooler C (1978). The structure of coping. Journal of Health and Social Behavior.

[ref-40] Phillips LS, Branch WT, Cook CB, Doyle JP, El-Kebbi IM, Gallina DL, Miller CD, Ziemer DC, Barnes CS (2001). Clinical inertia. Annals of Internal Medicine.

[ref-41] Prasad A, Shellito N, Alan Miller E, Burr JA (2023). Association of chronic diseases and functional limitations with subjective age: the mediating role of sense of control. Journals of Gerontology, Series B: Psychological Sciences and Social Sciences.

[ref-42] Schumann ME, Coombes BJ, Gascho KE, Geske JR, McDermott MC, Morrison EJ, Reynolds AL, Bernau JL, Gilliam WP (2022). Pain catastrophizing and pain self-efficacy mediate interdisciplinary pain rehabilitation program outcomes at posttreatment and follow-up. Pain Medicine.

[ref-43] Selvadurai D, Coleshill MJ, Day RO, Briggs NE, Schulz M, Reath J, Aung E (2024). Patient factors and health outcomes associated with illness perceptions in people with gout. Rheumatology.

[ref-44] Singh JA, Morlock A, Morlock R (2025). Gout flare burden in the United States: a multiyear cross-sectional survey study. ACR Open Rheumatology.

[ref-45] Strand V, Pillinger MH, Oladapo A, Yousefian C, Brooks D, Kragh N (2024). Patient experience with chronic refractory gout and its impact on health-related quality of life: literature review and qualitative analysis. Rheumatology and Therapy.

[ref-46] Tsiamalou P, Brotis AG, Vrekou E, Georgakopoulou VE, Papalexis P, Aravanatinou-Fatorou A, Tegousi M, Fotakopoulos G, Paterakis K (2023). The nurse’s role in managing gout in the modern era: a systematic review of the literature. Medical Intelligence.

[ref-47] Uritani D, Kasza J, Campbell PK, Metcalf B, Egerton T (2020). The association between psychological characteristics and physical activity levels in people with knee osteoarthritis: a cross-sectional analysis. BMC Musculoskeletal Disorders.

[ref-48] Van der Ven J, Van den Bemt BJF, Flendrie M, Vriezekolk JE, Verhoef LM (2025). Determinants of self-management behavior in gout: a scoping review. Arthritis Care and Research.

[ref-49] Walsh CP, Prior JA, Chandratre P, Belcher J, Mallen CD, Roddy E (2016). Illness perceptions of gout patients and the use of allopurinol in primary care: baseline findings from a prospective cohort study. BMC Musculoskeletal Disorders.

[ref-50] Wang Y, Chen Y, Qi Q, Song Y, Guo X, Ma L, Chen H (2025). The association between psychological capital and self-management behaviors in men with gout: a cross-sectional study in Southwest China. Patient Prefer Adherence.

[ref-51] Wang YB, Jin CZ (2024). Roles of traditional Chinese medicine extracts in hyperuricemia and gout treatment: mechanisms and clinical applications. World Journal of Gastroenterology.

[ref-52] Wang FJ, Zhang C, Cai MM, Zhang JQ, Wang HX (2024). Personal mastery and quality of life in patients with atrial fibrillation after radiofrequency ablation: the mediating role of health promoting behavior. Heart and Lung.

[ref-53] Watson L, Protheroe J, Mallen CD, Muller S, Roddy E (2024). Health literacy and gout characteristics in a primary care cohort. Rheumatology Advances in Practice.

[ref-54] Xiang L, Wang J, Li W, Ye H (2024). A study on the current situation and related factors of personal mastery in patients with chronic heart failure: a cross-sectional study. International Journal of General Medicine.

[ref-55] Younger JB (1991). A theory of mastery. ANS. Advances in Nursing Science.

[ref-56] Zeng TA, Xu M, Zhu H, Li FX, Duan XF, An WJ, Yang FY (2025). Relationship between mindfulness and self-control in deaf individuals: mediating role of inner peace and moderating role of life experience. BMC Psychology.

[ref-57] Zhou W, Zhu J, Guo J, Chen H, Zhang X, Gu Z, Zhou F, Dong C (2020). Health-related quality of life assessed by Gout Impact Scale (GIS) in Chinese patients with gout. Current Medical Research and Opinion.

[ref-58] Zimmer F, Draxler C, Debelak R (2023). Power analysis for the Wald, LR, score, and gradient tests in a marginal maximum likelihood framework: applications in IRT. Psychometrika.

