# Supplementary material for: The current status and influencing factors of personal mastery in patients with gout: a cross-sectional study
Source: PeerJ. 2026 Jun 8;14:e21393. doi: 10.7717/peerj.21393 (PMC13256057; doi:10.7717/peerj.21393)
Supplement: Supplemental Information 1 [file peerj-14-21393-s001.docx]

**Table S1 Personal Mastery Scale (PMS).**

| **Scale Items** | **Completely Disagree** | **Slightly Disagree** | **Somewhat Agree** | **Mostly Agree** | **Completely Agree** |
| --- | --- | --- | --- | --- | --- |
| 1. I feel helpless when dealing with life's difficulties. | 5 | 4 | 3 | 2 | 1 |
| 2. I can hardly change many important things in my life. | 5 | 4 | 3 | 2 | 1 |
| 3. I feel that my life is beyond my control. | 5 | 4 | 3 | 2 | 1 |
| 4. I have almost no control over what happens to me. | 5 | 4 | 3 | 2 | 1 |
| 5. I cannot solve the problems I encounter in my life. | 5 | 4 | 3 | 2 | 1 |
| 6. I can almost do anything I want to do. | 1 | 2 | 3 | 4 | 5 |
| 7. What my future will be like mainly depends on myself. | 1 | 2 | 3 | 4 | 5 |
