# Supplementary material for: The current status and influencing factors of personal mastery in patients with gout: a cross-sectional study
Source: PeerJ. 2026 Jun 8;14:e21393. doi: 10.7717/peerj.21393 (PMC13256057; doi:10.7717/peerj.21393)
Supplement: Supplemental Information 3 [file peerj-14-21393-s003.docx]

| **Codebook for Supplemental Data S1 Variables** | | |
| --- | --- | --- |
| Variable Name | Code (Value) | Description (Category) |
| **Gender** | 1 | Male |
|  | 2 | Female |
| **Age (years)** | 1 | 18~44 |
|  | 2 | 45~59 |
|  | 3 | 60~74 |
|  | 4 | ≥75 |
| **Education Level** | 1 | Primary school or below |
|  | 2 | Junior High School |
|  | 3 | High School |
|  | 4 | Junior college or above |
| **Monthly Income (RMB)** | 1 | <2000 |
|  | 2 | 2000~3999 |
|  | 3 | 4000~5999 |
|  | 4 | 6000~7999 |
|  | 5 | ≥8000 |
| **Residence** | 1 | Rural |
|  | 2 | Urban |
| **BMI(kg/m²)** | 1 | Normal (18.50~23.90) |
|  | 2 | Overweight (24.00~27.90) |
|  | 3 | Obesity (≥28.00) |
| **Uric acid** | 1 | ≤360.00 μmol/L |
|  | 2 | ＞360.00 μmol/L |
| **Duration of illness (years)** | 1 | <1 |
|  | 2 | 1~4 |
|  | 3 | 5~9 |
|  | 4 | 10~14 |
|  | 5 | ≥15 |
| **Number of attacks** | 1 | 0 |
|  | 2 | 1~3 |
|  | 3 | 4~6 |
|  | 4 | ≥7 |
| **Tophi** | 0 | No |
|  | 1 | Yes |
| **Family history** | 0 | No |
|  | 1 | Yes |
| **Comorbidities** | 0 | 0 |
|  | 1 | 1 |
|  | 2 | 2 |
|  | 3 | ≥3 |

****Note:****

****Personal mastery 1-7****: Items of the Personal Mastery scale.

****Mindfulness 1-15****: Items of the Mindfulness scale.

****Pain self-efficacy 1-10****: Items of the Pain Self-Efficacy scale.

****Total Scores (e.g., Personal mastery total, Mindfulness total, Pain self-efficacy total)****: Represent the sum of scores from their respective scale items. The specific Likert scale range (e.g., 1-5, 0-6) for each item should be confirmed by the original scales' scoring manuals.
