# Supplementary material for: The current status and influencing factors of personal mastery in patients with gout: a cross-sectional study
Source: PeerJ. 2026 Jun 8;14:e21393. doi: 10.7717/peerj.21393 (PMC13256057; doi:10.7717/peerj.21393)
Supplement: Supplemental Information 4 [file peerj-14-21393-s004.doc]

STROBE Statement—Checklist of items that should be included in reports of ***cross-sectional studies***

|  | Item No | Recommendation | Notes |
| --- | --- | --- | --- |
| **Title and abstract** | 1 | (*a*) Indicate the study’s design with a commonly used term in the title or the abstract | The title clearly states it is a "cross-sectional study." P1, L1-2. |
|  |  | (*b*) Provide in the abstract an informative and balanced summary of what was done and what was found | The abstract succinctly summarizes objectives, methods, key results, and conclusions.P1, L13-28. |
| Introduction | | |  |
| Background/rationale | 2 | Explain the scientific background and rationale for the investigation being reported | Provides a comprehensive background on gout, personal mastery, and the research gap. P2-4, L31-80 |
| Objectives | 3 | State specific objectives, including any prespecified hypotheses | Objectives are clearly stated at the end of the introduction. P4, L81-85 |
| Methods | | |  |
| Study design | 4 | Present key elements of study design early in the paper | The "The subject of the study" section immediately states the cross-sectional design and sampling method. P4-5, L87-103. |
| Setting | 5 | Describe the setting, locations, and relevant dates, including periods of recruitment, exposure, follow-up, and data collection | Setting: Shandong Gout Medical Center is named.  Dates: "Between October 17 and December 30, 2023" is provided. P4-5, L87-103. |
| Participants | 6 | (*a*) Give the eligibility criteria, and the sources and methods of selection of participants | Inclusion and exclusion criteria are clearly listed. The source and method are stated. P4-5, L87-103. |
| Variables | 7 | Clearly define all outcomes, exposures, predictors, potential confounders, and effect modifiers. Give diagnostic criteria, if applicable | Outcomes, predictors, and potential confounders are well-defined in the "Measures" section. P5-6,L104 -133. |
| Data sources/ measurement | 8* | For each variable of interest, give sources of data and details of methods of assessment (measurement). Describe comparability of assessment methods if there is more than one group | For each scale, the source, number of items, scoring, and range are provided. Cronbach's alpha from the literature and the current study are reported. P5-6,L104 -133. |
| Bias | 9 | Describe any efforts to address potential sources of bias | This study is a single-center investigation with a relatively homogeneous sample source, potentially introducing regional bias. P12, L285-L287. |
| Study size | 10 | Explain how the study size was arrived at | The sample size calculation based on the Kendall principle is clearly explained.P5, L97-100. |
| Quantitative variables | 11 | Explain how quantitative variables were handled in the analyses. If applicable, describe which groupings were chosen and why | Described in the "Data analysis" section. The categorization of continuous independent variables for the regression is shown in Table 3. |
| Statistical methods | 12 | (*a*) Describe all statistical methods, including those used to control for confounding | T-tests, ANOVA, Pearson's correlation, and multiple linear regression are described. P7, L143-149. |
|  |  | (*b*) Describe any methods used to examine subgroups and interactions | Not Applicable |
|  |  | (*c*) Explain how missing data were addressed | No missing variable data |
|  |  | (*d*) If applicable, describe analytical methods taking account of sampling strategy | Not Applicable |
|  |  | (*e*) Describe any sensitivity analyses | The results partially indicate the absence of collinearity. P8, L170-172. |
| Results | | |  |
| Participants | 13* | (a) Report numbers of individuals at each stage of study—eg numbers potentially eligible, examined for eligibility, confirmed eligible, included in the study, completing follow-up, and analysed | Table 1 shows the characteristics of the participants. A total of 234 patients with gout. P7, L153. |
|  |  | (b) Give reasons for non-participation at each stage | No missing variable data |
|  |  | (c) Consider use of a flow diagram | Not Applicable |
| Descriptive data | 14* | (a) Give characteristics of study participants (eg demographic, clinical, social) and information on exposures and potential confounders | Table 1 provides comprehensive demographic and clinical characteristics. |
|  |  | (b) Indicate number of participants with missing data for each variable of interest | No missing variable data |
| Outcome data | 15* | Report numbers of outcome events or summary measures | The mean and standard deviation for the main outcome and key predictors are reported in the text. Table 2. |
| Main results | 16 | (*a*) Give unadjusted estimates and, if applicable, confounder-adjusted estimates and their precision (eg, 95% confidence interval). Make clear which confounders were adjusted for and why they were included | Table 4 presents the adjusted estimates from the multiple linear regression. The variables adjusted for are clear. |
|  |  | (*b*) Report category boundaries when continuous variables were categorized | The coding for categorized variables is clearly shown in Table 3. |
|  |  | (*c*) If relevant, consider translating estimates of relative risk into absolute risk for a meaningful time period | Not Applicable |
| Other analyses | 17 | Report other analyses done—eg analyses of subgroups and interactions, and sensitivity analyses | The results partially indicate the absence of collinearity. P8, L168-170. |
| Discussion | | |  |
| Key results | 18 | Summarise key results with reference to study objectives | The first paragraph of the discussion summarizes the key finding and subsequent paragraphs discuss the influencing factors as per the objectives. P8-9, L180-197. |
| Limitations | 19 | Discuss limitations of the study, taking into account sources of potential bias or imprecision. Discuss both direction and magnitude of any potential bias | The "Study strengths and limitations" section thoroughly discusses the cross-sectional design, single-center bias, and gender imbalance. P12, L281-296. |
| Interpretation | 20 | Give a cautious overall interpretation of results considering objectives, limitations, multiplicity of analyses, results from similar studies, and other relevant evidence | The discussion provides a balanced interpretation, linking findings to theory and literature, while being mindful of the limitations. P8-12, L180-273. |
| Generalisability | 21 | Discuss the generalisability (external validity) of the study results | The limitations section directly addresses generalizability, noting the single-center setting. P12, L285-287. |
| Other information | | |  |
| Funding | 22 | Give the source of funding and the role of the funders for the present study and, if applicable, for the original study on which the present article is based | The "Funding" section states "The authors received no specific funding for this work." |

*Give information separately for exposed and unexposed groups.

**Note:** An Explanation and Elaboration article discusses each checklist item and gives methodological background and published examples of transparent reporting. The STROBE checklist is best used in conjunction with this article (freely available on the Web sites of PLoS Medicine at http://www.plosmedicine.org/, Annals of Internal Medicine at http://www.annals.org/, and Epidemiology at http://www.epidem.com/). Information on the STROBE Initiative is available at www.strobe-statement.org.
